# Association between Day-to-Day Pulsatility Index Change and Neurocognitive Outcomes in Pediatric Traumatic Brain Injury

**DOI:** 10.1089/neur.2022.0035

**Published:** 2022-09-01

**Authors:** Jeremy Jordan, Sigrid Ladores, Michele Kong, Tedra Smith, Peng Li, Karin Reuter-Rice

**Affiliations:** ^1^ School of Nursing, School of Medicine, University of Alabama at Birmingham, Birmingham, Alabama, USA.; ^2^ Pediatric Critical Care Medicine, Children's of Alabama, Birmingham, Alabama, USA.; ^3^ School of Nursing, Duke University, Durham, North Carolina, USA.; ^4^ Department of Pediatrics, School of Medicine, University of Alabama at Birmingham, Birmingham, Alabama, USA.

**Keywords:** cerebrovascular circulation, patient outcomes, pediatrics, transcranial Doppler ultrasound, traumatic brain injury

## Abstract

Traumatic brain injury (TBI) remains a significant cause of morbidity and mortality in children despite advances in prevention and mitigation strategies. Transcranial Doppler (TCD) ultrasound measures cerebral arterial circulation and allows for the calculation of pulsatility indices (PIs), which provides an assessment of cerebral blood flow changes. Yet, the use of PIs in children with TBI is not well understood. In this study, we defined the day-to-day (DTD) PI change of the anterior cerebral circulation and describe its relationship with injury characteristics and neurocognitive outcomes in children with TBI. A prospective observational parent study of 42 children, 2 months to 15 years of age, with mild or moderate-severe TBI who had serial TCDs provided data for this analysis. Both the mean and variation of DTD PI change were evaluated in the context of injury severity, injury sidedness, and neurocognitive outcome. In those with a unilateral injury, a larger mean DTD PI change in both the injured and uninjured side was found in those with a worse Glasgow Outcome Scale-Extended Pediatrics score at discharge. A larger variation in PI was associated with a worse neurocognitive outcome, irrespective of injury severity. Therefore, the mean and variation of DTD PI change may serve as a potential cerebral vascular biomarker of ongoing secondary injury. The use of PI measurements in the monitoring of children with TBI may provide clinicians with new diagnostic and prognostic insights to inform therapeutic interventions and recovery strategies. However, a larger prospective study is needed to confirm these findings and elucidate potential mechanistic links between DTD PI and clinical outcome measures. To our knowledge, this study is the first of its kind to evaluate the use of PI changes in cerebral vasculature in pediatric TBI patients admitted to the hospital.

## Introduction

Traumatic brain injury (TBI) remains a significant cause of morbidity and mortality in the United States, with >2.8 million injuries occurring annually.^1^ Children account for >475,000 of these persons.^2^ TBI is the cause of one third of all injury-related deaths in children <19 years of age in the United States, totaling >7400 children.^3^ Children who survive TBI are at increased risk for physical disabilities, mental health disorders, developmental delay, and neurological impairment.^4,5^ The economic burden of pediatric TBI is significant, at an average lifetime cost of $600,000–$1,875,000.^6^

A TBI occurs when there is an injury to the skull and brain by either direct or indirect external mechanical force, which results in two injury phases: the primary injury phase followed by the secondary injury phase.^7–9^ The primary injury phase occurs at the time of injury impact and is the result of mechanical forces that structurally disrupt and/or damage the brain, which include axonal tearing, hemorrhage, compression, and contusions.^10–12^ Secondary injury follows the primary injury and occurs over minutes to days and results in hypoxia, altered cerebral blood flow, metabolic dysregulation, and neuroinflammation.^10–12^ Therefore, assessment of injury in the secondary injury phase is critical to promote optimal neuroprotection that can lead to improved outcomes. Non-invasive bedside-based neuromonitoring options to detect potential ongoing brain injury remain limited in children with TBI.

Transcranial Doppler (TCD) ultrasonography is a non-invasive, portable, safe, and inexpensive imaging modality, compared to previous modalities such as xenon enhanced computerized tomography, which allows clinicians to evaluate cerebral circulation.^13,14^ Although TCD remains an emerging area of research in pediatric TBI, it has been used to evaluate the anterior cerebral circulation through the middle cerebral artery (MCA).^2,15–17^ Evaluation of cerebral blood flow velocity (CBFV) in MCAs can aid in the diagnosis of hyperemia and cerebral vasospasm.^2,15^ In preliminary studies, alterations in CBFV from baseline are associated with poorer neurological outcomes in children with TBI.^2,18^ The use of TCD to measure CBFV includes the Gosling pulsatility index (PI). The PI is a hemodynamic index, calculated as the difference between systolic and diastolic flow velocities divided by the mean velocity [CBFV_sys_ – CBFV_dia_)/CBFV_mean_].^19^ There have been no definitive normal and abnormal values formally established for PI in the pediatric TBI population. However, early research indicated that PI may be an important measurement to further understand the ongoing secondary injury phase of a TBI.

Alterations in PI have been associated with intracranial pathology, indicating that PI may be a marker of cerebrovascular resistance as a result of vasospasm, hyperemia, or changes in intracranial pressure (ICP).^20–22^ Recent case studies have demonstrated the potential use of TCD in the clinical management of pediatric patients with intracranial pathology.^23^ Therefore, the use of TCD as a non-invasive bedside neuromonitor allows the clinician to determine the PI of the cerebral circulation and may provide insights into the secondary injury phase and physiological state of the brain after TBI.^20,24,25^

**FIG. 1.**
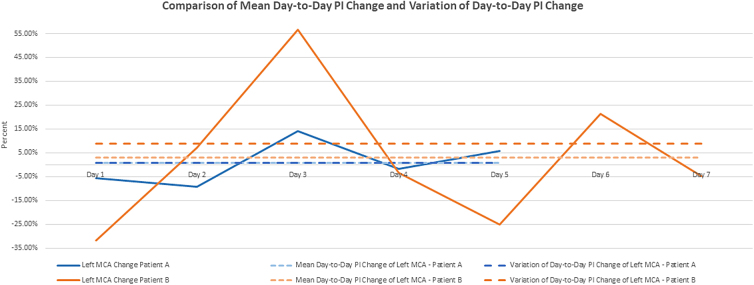
Comparison of mean day-to-day PI change and variation of day-to-day PI change. MCA, middle cerebral artery; PI, pulsatility index.

In existing research, the PI is typically analyzed as a single measurement in time.^26–29^ Day-to-day (DTD) PI change is a novel concept, first reported by Jordan and Reuter-Rice, that describes the change in the PI measurement from one day to the subsequent day (e.g., the change in the PI measurement from post-injury day 1 to post-injury day 2, the change from day 2 to day 3, and so on).^30^ This concept is operationalized by calculating the percent change of the PI measurement from one day to the next day [(PI_day2_ – PI_day1_) / PI_day1_]. Jordan and Reuter-Rice found that children with TBI who had a higher mean DTD PI change had a worse Glasgow Outcome Scale-Extended Pediatrics (GOS-E Peds) score.^30^ However, the mean DTD PI change, used by Jordan and Reuter-Rice, only calculates the mean difference and does not describe the variability of the PI measurements (i.e., how far each PI measurement is from the mean value). For example, Figure 1 demonstrates the DTD PI change for 2 different participants. Both participants have similar mean DTD PI change. However, the variation of the DTD PI change is larger in 1 participant. Thus, variation of change may be another predictor of neurocognitive outcomes for children with TBI in addition to the mean change.

Additionally, the original analysis focused on the descriptive features of DTD PI change only.^30^ The purpose of this article is to explore the relationship between mean DTD PI change, variation of DTD PI change, injury severity, and neurocognitive outcomes in children with TBI. To further explore the importance of these findings, an inferential statistical analysis is proposed.

## Methods

### Parent and daughter studies

Subsequent to institutional review board approval and upon parental consent, the parent study recruited 60 children (ages 10 days to 15 years) who were admitted to a tertiary-care level 1 trauma center for a TBI from December 2012 through August 2015 (National Institutes of Health/National Institute of Nursing Research, 1P30-NR014139; Robert Wood Johnson Foundation, 71244). The prospective exploratory study measured daily CBFV and PIs using TCD, cognitive, and functional outcomes at time of discharge. All participants were previously healthy without any known past history of TBI and were enrolled into the study within 24 h of admission. Enrollment criteria included: 1) admission to hospital; 2) age of 5 days to 15 years; 3) a diagnosis of TBI; 4) ability to undergo adequate TCD; and 5) English or Spanish speaking. Children were excluded from participating if there was a previously diagnosed significant neurodevelopmental delay or a diagnosis of non-traumatic intracranial hemorrhage. These children were excluded because a previous baseline neurocognitive function was unavailable to establish a comparison and they had a mechanism of injury and intracranial pathophysiology that might alter cerebral hemodynamics in a way that differed from that of the desired study population.

For this study, after institutional review board approval and a data transfer agreement were completed, a deidentified database of the recruited 60 children was screened. Inclusion criteria, including the inclusion criteria for the parent study, were consecutive MCA PI measurements. This resulted in 43 children, 2 months to 15 years of age, being eligible for inclusion. Data collection included documented demographic information, TCD examinations, and outcome measures. Injury severity, based on the Glasgow Coma Scale (GCS) for infants and children, was determined at time of evaluation in the emergency department. The GCS is a universally accepted, standardized measure used for grading TBI severity. The GCS is a 15-point clinical scale that evaluates three dimensions: eye opening; best verbal response; and best motor response. TBI severity is categorized as: mild = 13– to 15; moderate = 9–12; and severe = 3–8.^31^

The outcome measure used in the parent and therefore this study was the GOS-E Peds. The GOS-E Peds is a validated, developmentally appropriate, structured instrument that measures functional and neurocognitive outcomes.^32^ It uses an 8-point scale, with a score of 1 indicating upper good recovery through a score of 8 indicating death.^32^ In this study the GOS-E Peds was used to measure functional outcomes at the time of discharge.

### Transcranial Doppler ultrasonography

Participants in the parent study underwent an initial TCD within 24 h of enrollment to the study and daily TCD examinations, which continued through hospital day 8, discharge, or death. The healthcare team was blinded to the TCD results, and the TCDs were not used to direct care. Because TCDs are not considered standard of care in pediatric TBI, no set number of missed TCDs disqualified participants from the parent study. The bedside TCD was performed by certified sonographers from the Department of Neurodiagnostic Studies. Sonographers used a commercially available TCD ultrasonography unit with a 2-MHz pulsed probe (Sonara Digital TCD; CareFusion, Middleton, WI). The study protocol followed the method described by Aaslid and colleagues, where the anterior circulation was measured by insonating the bilateral MCAs every 2–5 mm at standard depths.^13^ TCD ultrasounds were interpreted by a blinded certified TCD neurologist who determined all TCD interpretations.

### Statistical analysis

Data on the 43 children were downloaded from REDCap and analyzed using R software (version 4.0; R Core Team, 2020). Descriptive statistics were used to describe the sample characteristics, mean DTD PI change, variation of DTD PI change (described below), and outcome measurements. Because of the small sample size, children were grouped as either mild (GCS 13–15) or moderate/severe (GCS 3–12). All children were included in the first analysis; however, 1 child was excluded in the final analysis because, as an outlier, when included in the analysis the mean DTD PI change and variation of DTD PI change resulted in a 400 × increase in the group mean DTD PI change and 2800 × increase in the group variation of DTD PI change (Table 1). Therefore, the final reported analysis includes a total of 42 children.

**Table 1.** Mean Day-to-Day PI Change and Variation of Day-to-Day PI Change

	Mean day-to-day PI change Mean ± SD	Variation of day-to-day PI change Mean ± SD
Bilateral/global injury without outlier		
Left MCA	0.0123 ± 0.1155	0.1076 ± 0.1050
Right MCA	–0.0481 ± 0.1459	0.0558 ± 0.0513
Bilateral/global injury with outlier		
Left MCA	0.434 ± 1.691	28.5987 ± 98.6959
Right MCA	–0.0436 ± 0.1421	0.0555 ± 0.0487

PI, pulsatility index; MCA, middle cerebral artery; SD, standard deviation.

To define and describe mean DTD PI change and variation of DTD PI change, we provide the following steps:

- Step 1: DTD PI change is defined as the difference in PI measurement from one day to the next and is calculated using the following formula (using day 1 and day 2 as an example):
- *(PI_day2_ – PI_day1_) / PI_day1_ = day-to-day PI change*
- The DTD PI changes for other continuous days were calculated similarly.
- Step 2: We averaged these values to derive their mean DTD PI change during the hospital stay. This value represents the mean difference between each consecutive day's measurement.^30^
- Step 3: We then determined the variation of DTD PI change, defined as the dispersion of PI measurements from their mean values by using the variation function. Values were calculated for both MCAs in each participant. We used the left MCA PI and right MCA PI to describe sidedness and determine their relationship to injury severity and neurocognitive outcome. Injury sidedness was determined by the participants' initial head computed tomography and was reported by the parent study as left, right, or global/bilateral.

The PI for this group was not normally distributed, therefore non-parametric tests were used. The mean DTD PI change and variation of DTD PI change for the ipsi- and contralateral MCAs in participants with a unilateral injury as well as both the left and right MCA in participants with a bilateral or global injury were compared using a Wilcoxon signed-rank test. Additionally, the mean DTD PI change and variation of DTD PI change were evaluated in the context of severity using a Wilcoxon rank-sum test. Last, eight general linear regression models were created to explore the effects of mean DTD PI change and variation of DTD PI change of each MCA on the GOS-E Peds score at discharge, controlling for injury severity. The adjusted *R*^2^ and Bayesian information criterion (BIC) were used to evaluate the goodness of fit for each model, where larger adjusted *R*^2^ and smaller BIC indicated better model fit.

### Institutional review board waiver

This study is judged exempt, Category 4, with no continuing review from the Institutional Review Board at the University of Alabama at Birmingham (IRB-300001715)

## Results

Children in the study sample were majority white (59.5%), male (57.1%), and had mild TBI (59.5%; Table 2). The average number of TCD evaluations was 3.8 for each child. Children with mild injury were hemodynamically stable whereas the moderate/severe TBIs were managed based on the second edition of the pediatric TBI management guidelines to maintain stable hemodynamic and cerebrodynamic stability.^33^ Of the sample, only 9 patients had ICP monitoring, thereby limiting the opportunity to compare ICP to PI measurements.

**Table 2.** Individual Characteristics and Demographics

Characteristic	Sample* n* = 42 (%)
Sex	
Male	24 (57.1)
Female	18 (42.9)
Race/ethnicity	
White/Caucasian	25 (59.5)
Black/African American	14 (33.3)
American Indian or Alaskan Native	1 (2.4)
Asian	1 (2.4)
Other	1 (2.4)
Age	
<6 months	10 (23.8)
6 months to 3 years	9 (21.4)
4–6 years	6 (14.3)
7–15 years	17 (40.5)
Severity of injury	
Mild	25 (59.5)
Moderate/severe	17 (40.5)
Sidedness of injury	
Right side	16 (38.1)
Left side	11 (26.2)
Bilateral/global	15 (35.7)
GOS-E Peds at discharge	
Upper good	9 (21.4)
Lower good	13 (31.0)
Upper moderate	5 (11.9)
Lower moderate	4 (9.5)
Upper severe	3 (7.1)
Lower severe	6 (14.3)
Dead	2 (4.8)
Mechanism of injury	
Non-accidental trauma	15 (35.7)
Fall	14 (33.3)
Motorized vehicle collision	5 (11.9)
Other	8 (19)

GOS-E Peds, Glasgow Outcome Scale-Extended Pediatrics.

In children with a unilateral injury, both the mean DTD PI change (0.0615 ± 0.1953) and variation of DTD PI change (0.1331 ± 0.3428) were higher in the uninjured side when compared to the injured side (0.0027 ± 0.0953 and 0.0391 ± 0.0391, respectively). Whereas for children with bilateral/global injuries, the mean DTD PI change (−0.0481 ± 0.1459) in the right side was found to be greater than the left (0.0123 ± 0.1155), whereas the variation of DTD PI change (0.1077 ± 0.1050) in the left side was found to be larger than the right (0.0558 ± 0.0513; Table 3).

**Table 3.** Mean Day-to-Day PI Change and Variation of Day-to-Day PI Change by Sidedness

	Middle cerebral artery Mean ± SD		Mean of the difference^*^ Mean (95% CI)
Unilateral injury	Injured-side MCA	Uninjured-side MCA	
Mean day-to-day PI change	0.0027 ± 0.0953	0.0615 ± 0.1953	–0.0588 (−0.0819, 0.0094)
Variation of day-to-day PI change	0.0391 ± 0.0391	0.1331 ± 0.3428	–0.094 (−0.0717, 0.0116)
Bilateral/global injury	Left MCA	Right MCA	
Mean day-to-day PI change	0.0123 ± 0.1155	–0.0481 ± 0.1459	0.0604 (−0.0113, 0.1422)
Variation of day-to-day PI change	0.1077 ± 0.1050	0.0558 ± 0.0513	0.0518 (−0.0040, 0.1436)

^*^ Mean of the differences is calculated for participants with both measurements.

PI, pulsatility index; MCA, middle cerebral artery; SD, standard deviation; CI, confidence interval.

In the unilateral injury group, participants with a moderate/severe injury had, on average, a higher mean DTD PI change of the injured side and both a higher mean and variation of DTD PI of the uninjured side. In the bilateral/global injury group with moderate/severe injury, the mean DTD PI change of the left side and right side were higher, whereas the variation of DTD PI change of the right side was higher (Table 4).

**Table 4.** Mean Day-to-Day PI Change and Variation of Day-to-Day PI Change by Severity Group

	Mild injury group ****(***n* = 25) Mean ± SD	Moderate/severe ****(***n* = 17) Mean ± SD	Difference of the means Mean (95% CI)
Unilateral injury			
Injured side mean day-to-day PI change	0.0109 ± 0.1069	–0.0168 ± 0.0615	0.0277 (−0.0462 to 0.0970)
Injured side variation of day-to-day PI change	0.0316 ± 0.0313	0.04940 ± 0.04816	–0.0178 (−0.0722 to 0.0167)
Uninjured side mean day-to-day PI change	0.0726 ± 0.2131	0.0350 ± 0.1541	0.0376 (−0.1025 to 0.1262)
Uninjured side variation of day-to-day PI change	0.0470 ± 0.0559	0.2515 ± 0.5197	–0.2045 (−0.1055 to 0.0106)
Bilateral/global injury			
Left MCA mean day-to-day PI change	0.0128 ± 0.1228	0.0119 ± 0.1180	0.0009 (−0.1473 to 0.1301)
Right MCA mean day-to-day PI change	–0.0523 ± 0.1605	–0.0452 ± 0.1453	–0.0071 (−0.1705 to 0.1793)
Left MCA variation of day-to-day PI change	0.0107 ± 0.0118	0.1292 ± 0.1044	–0.1185 (−0.2877 to 0.0016)
Right MCA variation of day-to-day PI change	0.0116 ± 0.0117	0.0669 ± 0.0517	–0.0553 (−0.1647 to 0.0182)

PI, pulsatility index; MCA, middle cerebral artery; SD, standard deviation; CI, confidence interval.

To explore the associations among mean DTD PI change and variation of DTD PI change on GOS-E Peds score at discharge while controlling for severity, eight regression models are evaluated (Tables 5 and 6**)**. For those with a unilateral injury, models 1 and 3 best fit the data, with an adjusted *R*^2^ of 0.58 and 0.58 and a BIC of 90.8 and 69.5, respectively (Table 5**)**. Model 1 demonstrates that having a moderate/severe injury had a large effect size (standardized beta coefficient, β = 0.78; 95% confidence interval [CI], −0.16 to 1.72) on GOS-E Peds score at discharge, whereas an elevated mean DTD PI change of the uninjured side had a smaller effect size (β = 0.15; 95% CI, −2.1 to 2.4) on GOS-E Peds score at discharge. Model 3 also demonstrates that having a moderate/severe injury had a large effect size (β = 0.71; 95% CI, −0.54 to 1.95) and that having an elevated variation of DTD PI change on the uninjured side had a small effect size (β = 0.2; 95% CI, −1.64 to 2.04) on GOS-E Peds score at discharge. In both models, the positive standardized beta coefficients indicated that a moderate/severe injury, elevated mean DTD PI change, and elevated variation of DTD PI change are associated with a higher GOS-E Peds score at discharge (i.e., the worse outcome).

**Table 5.** Linear Models for Unilateral Injury, Mean Day-to-Day PI Change, Variation of Day-to-Day PI Change, and GOS-E Peds Score at Discharge

Model		Adj* R*^2^	F test* p *value	BIC
1.	GOS-E Peds_T1∼Severity_Group+Uninjured_MCA_MeanChng	0.58	<0.001	90.8
2.	GOS-E Peds_T1∼Severity_Group+Injured_MCA_MeanChng	0.55	<0.001	92.3
3.	GOS-E Peds_T1∼Severity_Group+Uninjured_MCA_Var	0.58	<0.001	69.5
4.	GOS-E Peds_T1∼Severity_Group+Injured_MCA_Var	0.54	0.001	71.3

PI, pulsatility index; GOS-E Peds, Glasgow Outcome Scale-Extended Pediatrics; BIC, Bayesian information criterion.

**Table 6.** Linear Models for Bilateral/Global Injury, Mean Day-to-Day PI Change, Variation of Day-to-Day PI Change, and GOS-E Peds Score at Discharge

Model		Adj* R*^2^	F test* p *value	BIC
5.	GOS-E Peds_T1∼Severity_Group+Left_MCA_MeanChng	0.52	0.005	65.1
6.	GOS-E Peds_T1∼Severity_Group+Right_MCA_MeanChng	0.53	0.004	65.0
7.	GOS-E Peds_T1∼Severity_Group+Left_MCA_Var	0.39	0.057	50.8
8.	GOS-E Peds_T1∼Severity_Group+Right_MCA_Var	0.45	0.051	46.3

PI, pulsatility index; GOS-E Peds, Glasgow Outcome Scale-Extended Pediatrics; BIC, Bayesian information criterion.

The models for the bilateral/global injury group had lower adjusted *R*^2^ values. However, model 6 had the best fit, with an adjusted *R*^2^ of 0.53 and a BIC of 65. This model demonstrates that having a moderate/severe injury had a large effect size (β = 0 .76; 95% CI, −1.12 to 2.65) and that having an elevated mean DTD PI change of the right MCA had a small effect size (β = 0.09; 95% CI, −6.47 to 6.65) on GOS-E Peds score at discharge. Similarly, the positive standardized beta coefficients indicate that a more severe injury and an elevated mean DTD PI change are associated with a higher GOS-E Peds score at discharge, that is, the worse outcome for children with bilateral/global injury (Table 6).

## Discussion

In a group of 42 pediatric participants with TBI who had daily TCD measurements of their bilateral MCAs, the mean of DTD PI change and variation of DTD PI change were evaluated in the context of injury severity and the GOS-E Peds score at discharge. Our study demonstrated a difference in mean DTD PI change and variation of DTD PI change based on the side of the injury. The mean and variation of DTD PI change were larger in the uninjured side MCA in children with a unilateral injury. This difference could partially represent early evidence of a compensatory mechanism of the cerebral vasculature in the uninjured side, which is observed in cerebral autoregulation after TBI.^34,35^ This difference may also suggest autoregulatory dysfunction in the injured side, a known pathophysiological phenomenon after TBI.^34,35^ A higher mean and/or variation of DTD PI change in the uninjured side may represent a pathological change to maintain low ICP and/or appropriate cerebral perfusion pressure (CPP). In children with a bilateral/global injury, the sample was not large enough to detect a significant difference in DTD PI change between the left and right sides.

In children with a unilateral injury, a larger mean DTD PI change in both the injured and uninjured side was found in those with a worse GOS-E Peds score at discharge. This finding suggests that changes in PI, as opposed to a single PI measurement, may potentially be a useful clinical measure in prognostication after an isolated TBI in children, although further research is needed to confirm this relationship. Additionally, in this cohort, a larger variation in PI was associated with a worse neurocognitive outcome, irrespective of injury severity. Therefore, the mean and variation of DTD PI change may serve as a potential cerebral vascular biomarker of ongoing secondary injury. The use of PI measurement in the monitoring of children with TBI may therefore provide clinicians with new diagnostic and prognostic insights to inform therapeutic interventions and recovery strategies.

By evaluating change in PI over time rather than at one discrete time point, our findings suggest that it may be clinically useful to evaluate PI values serially. Additionally, a larger mean DTD PI change and larger variation of DTD PI change were found in children with a moderate/severe TBI when compared to those with a mild injury. Whereas TCD is still an emerging area of research in pediatric TBI, evaluations of DTD PI change are conceptually consistent with research findings in adult TBI, which demonstrate an association between increased PI and intracranial pathological changes.^20^

Additionally, the utility of TCD in pediatric TBI is further supported, which is consistent with the recently released multi-disciplinary consensus statement recommending the routine use of TCD in pediatric ICU patients who are at risk for alterations in cerebral hemodynamics.^36^ In particular, the standardization of TCD protocols, interpretation, and reporting will be of benefit to future research of mean and variation of DTD PI change in children with alterations in cerebral hemodynamics.

Although the small, single-site sample size of 42 participants limits generalizability, the evaluation of mean and variation of DTD PI change of the MCAs was associated with more severe injury as well as a worse GOS-E Peds score at discharge. Although this study provides that an early signal of change in PI over time may be of significant value, additional research with a larger study sample is needed to further confirm these relationships and further explore potential mechanisms or physiological underpinnings that could lead to therapeutic targets. This additional research should include a larger sample with significant representation of mild, moderate, and severe injury as well as mechanisms of injury. Research including other physiological data, such as invasive ICP measurement, CPP, partial pressure of oxygen in the brain, carbon dioxide levels, blood pressure, and more frequent TCD and PI measurement, is needed to further our understanding of alteration in PI in children with TBI.

## Conclusion

To our knowledge, this study was a first of its kind to evaluate the use of PI changes in cerebral vasculature in pediatric TBI patients admitted to the hospital. Our exploratory findings suggest that mean and variation of DTD PI changes may serve as an early biomarker of ongoing secondary injury that can result in poorer neurocognitive/functional outcomes. These results further support the continued research of the role of TCD in the diagnosis, management, and prognostication of children with TBI.

## Abbreviations Used

BIC: Bayesian information criterion
CBFV: cerebral blood flow velocity
CI: confidence interval
CPP: cerebral perfusion pressure
DTD: day-to-day
GCS: Glasgow Coma Scale
GOS-E Peds: Glasgow Outcome Scale-Extended Pediatrics
ICP: intracranial pressure
MCA: middle cerebral artery
PI: pulsatility index
TBI: traumatic brain injury
TCD: transcranial Doppler ultrasonography
